# Disparities in Kaposi sarcoma incidence and survival in the United States: 2000-2013

**DOI:** 10.1371/journal.pone.0182750

**Published:** 2017-08-22

**Authors:** Kathryn E. Royse, Firas El Chaer, E. Susan Amirian, Christine Hartman, Susan E. Krown, Thomas S. Uldrick, Jeannette Y. Lee, Zachary Shepard, Elizabeth Y. Chiao

**Affiliations:** 1 Center of Innovations in Quality, Effectiveness and Safety, Michael E. DeBakey Veterans Affairs Medical Center, Houston, Texas, United States of America; 2 Department of Medicine, Section of Infectious Diseases, Baylor College of Medicine, Houston, Texas, United States of America; 3 Department of Infectious Diseases, Infection Control & Employee Health, The University of Texas MD Anderson Cancer Center, Houston, Texas, United States of America; 4 Dan L. Duncan Cancer Center, Baylor College of Medicine, Houston, Texas, United States of America; 5 Department of Pediatrics, Baylor College of Medicine, Houston, Texas, United States of America; 6 AIDS Malignancy Consortium, New York, New York, United States of America; 7 HIV and AIDS Malignancy Branch, National Cancer Institute, National Institutes of Health, Bethesda, Maryland, United States of America; 8 Department of Biostatistics, University of Arkansas for Medical Sciences, Little Rock, Arkansas, United States of America; 9 Department of Undergraduate Education, Baylor College of Medicine, Houston, Texas, United States of America; Fudan University, CHINA

## Abstract

**Objective:**

Geographic and racial disparities may contribute to variation in the incidence and outcomes of HIV-associated cancers in the United States.

**Method:**

Using the Surveillance, Epidemiology, and End Results (SEER) database, we analyzed Kaposi sarcoma (KS) incidence and survival by race and geographic region during the combined antiretroviral therapy era. Reported cases of KS in men from 2000 to 2013 were obtained from 17 SEER cancer registries. Overall and age-standardized KS incidence rates were calculated and stratified by race and geographic region. We evaluated incidence trends using joinpoint analyses and calculated adjusted hazard ratios (aHR) for overall and KS-specific mortality using multivariable Cox proportional hazards models.

**Results:**

Of 4,455 KS cases identified in men younger than 55 years (median age 40 years), the annual percent change (APC) for KS incidence significantly decreased for white men between 2001 and 2013 (APC -4.52, *p* = 0.02). The APC for AA men demonstrated a non-significant decrease from 2000–2013 (APC -1.84, *p* = 0.09). Among AA men in the South, however, APC has significantly increased between 2000 and 2013 (+3.0, *p* = 0.03). In addition, compared with white men diagnosed with KS during the same time period, AA men were also more likely to die from all causes and KS cancer-specific causes (aHR 1.52, 95% CI 1.34–1.72, aHR 1.49, 95% CI 1.30–1.72 respectively).

**Conclusion:**

Although overall KS incidence has decreased in the U.S., geographic and racial disparities in KS incidence and survival exist.

## Introduction

Kaposi sarcoma (KS), a cancer of endothelial cells, is etiologically linked to human herpesvirus 8 (HHV-8)[1], and highly associated with immunosuppression. In the U.S., the primary types of KS are: Classic KS, which primarily affects individuals over the age of 70[2], post-transplant KS[3], and epidemic, or HIV-associated KS. Individuals with advanced HIV disease have a risk of KS that is several thousand times the risk of the general population[4], leading to a global increase in the incidence of KS over the past 30 years[1, 5]. In particular, in parallel with the United States (U.S.) HIV epidemic, the incidence of KS in the U.S. peaked in the late 1980s at an age-standardized incidence rate of 33.3 per 100 000 person-years, and subsequently decreased in the late 1990s with the increased availability of combination antiretroviral therapy (ART)[6].

Although KS incidence has decreased in the U.S., AIDS-associated KS incidence in the U.S. varies by HIV geography and socioeconomic status[7]. For example, data from the International Collaboration on HIV and Cancer showed higher age-adjusted incidence rates for both HIV-related and classic KS in U.S. metropolitan areas than in rural areas[5]. More recently, a Surveillance, Epidemiology, and End Results (SEER) study analyzing the incidence of all cancers in the U.S. from 2005 through 2009 showed that KS incidence was positively associated with geographic areas of higher poverty[8]. However, there have been no studies evaluating the impact of race and geographic region on recent trends in likely AIDS-associated KS incidence.

Although African Americans (AA) comprised only 12% of the total U.S. population, they accounted for 50% of new HIV diagnoses in 2014[9]. Recently, southern states have lagged behind the rest of the U.S. in HIV testing and access to treatment[10]. Furthermore, death rates among people living with HIV vary geographically between states from 7.9 per 1000 HIV positive individuals in Vermont to as high as 30.8 per 1000 HIV positive individuals in Louisiana[10]. Thus, despite the nationwide progress seen in HIV care, disparities in access to HIV and other health care services between southern U.S. states and the rest of the country may impact the incidence and outcomes of KS.

To evaluate the potential effects of these regional and racial disparities on trends in KS incidence and mortality, we used SEER data to evaluate changes in the incidence and survival of people diagnosed with KS in the U.S. during the ART era.

## Materials and methods

### Data sources and patients

The SEER Program is considered the quality standard among worldwide cancer registries and is the only registry in the U.S. that tracks population-based staging and stage-dependent cancer survival data[11]. Though the SEER database has a higher population of foreign-born individuals than the U.S. population in general, it is similar to the general U.S. population with respect to poverty and education levels[8]. It includes approximately 28% of the U.S. population: 25% of whites, 26% of AAs, 38% of Hispanics, 44% of American Indians and Alaska Natives, 50% of Asians, and 66% of Hawaiian/Pacific Islanders[12]. SEER provides data on date of diagnosis, patient demographics, follow-up time, cause-specific and overall mortality, and has an estimated report completeness of >97%[13].

KS incidence and survival data for men diagnosed between 2000 and 2013 were obtained from 17 population-based cancer registries that participate in SEER[11]. We excluded the Louisiana registry because Hurricane Katrina significantly disrupted the reporting of data in that registry in 2005. Incidence and survival were compared between geographic regions as defined by the U.S. Census (South, West, Midwest, and Northeast) and by race. The South was defined as Atlanta, Rural Georgia, Greater Georgia, and Kentucky; the West included Hawaii, Alaska, Greater California, Los Angeles, San Jose-Monterey, San Francisco-Oakland, Seattle-Puget Sound, Utah, and New Mexico; the Northeast included Connecticut and New Jersey; and the Midwest included Detroit and Iowa.

### Classification of AIDS-associated Kaposi sarcoma

Because the SEER registry does not include HIV status or medication administration records, we estimated AIDS-related KS based on previously used methods[5, 14, 15]. Shiels et al found that while 81.6% of all KS cases in the U.S. were related to AIDS, over 94% of cases in individuals under the age of 60 had AIDS[16]. Additionally, 91% of KS cases in men and 100% of cases in women *older* than 55 years were due to classic, non-AIDS related KS[15]. Thus, we first evaluated crude incidence rates by age groups at time of diagnosis (20–29, 30–39, 40–54, and 55+). Subsequently, we then limited our analysis to men under 55 as those 55 years or older because this group was more likely to have classic, non-AIDS related, KS[16]. Women were also excluded because of the low numbers of cases in certain geographic subgroups. All cases were coded by the International Classification of Disease for Oncology as KS (ICD-O-9140/03).

### Study population

From SEER data, we abstracted only men diagnosed with incident KS. The time-period for KS diagnosis was from January 1, 2000 with all follow-up ending December 31, 2013. For follow-up calculations, we restricted our analysis to individuals whose KS diagnosis was either their only or first of multiple primary in-situ or malignant lifetime diagnosis, based on federally required annual reporting. For survival-specific analyses, we also excluded KS patients whose initial diagnosis was found either on death certificate (n = 13), at autopsy (n = 16), patients who were not under active follow-up (n = 29), or patients who had no or unknown microscopic confirmation of their cancer or death certificate only (n = 664). Patients with unknown or missing cause of death also were excluded from survival estimates (<1% of all KS diagnosis).

### Statistical analysis

Race information on incident KS patients was derived and grouped into white, African American, and other patients comprising Asians, American Indians/Alaska Natives, and Pacific Islanders. Age was recorded at KS diagnosis, age-standardized incidence rates, rate ratios and 95% confidence intervals were calculated by geographic region, race and age group. Demographics were summarized and plotted to compare trends descriptively using joinpoint regression analysis[17]. The joinpoint regression model fits a series of joined straight lines on a log scale to trends from age-adjusted rates based on the 2000 U.S. Census population. The magnitude of these trends from changing time-periods are described by the change in annual percent change (APC), which is the slope of this line segment (*p*<0.05)[18]. We compared KS incidence in age groups (20–29, 30–39, 40–54, and 55+), and after excluding men 55+, then calculated age-adjusted trends in KS incidence using the magnitude of change in the APC for KS incidence from 2000–2013 based on region, race, and these combinations. Each joinpoint indicated if there was a statistically significant change in the magnitude of the trend (APC)[18]. Joinpoint regression analyses were performed using statistical software from the United States National Cancer Institute Surveillance, Epidemiology, and End Results Program. (Joinpoint Regression Program, Version 4.3.1.0—April 2016; Statistical Methodology and Applications Branch, Surveillance Research Program, National Cancer Institute.)

In the survival analyses, we calculated endpoints defined with overall mortality, and SEER-cancer specific survival (CSS). CSS is part of the SEER cause-specific death classification variable algorithm that considers cause of death in combination with the tumor sequence, first malignant diagnosis, and comorbidities, including AIDS, to capture deaths attributable to a specific cancer diagnosis[19]. Because KS is more likely to occur in HIV infected individuals, CSS may more accurately reflect the attributable mortality[19–21]. Survival curves for those incident KS diagnoses included in the survival analyses used month and year of death to construct and visualize differences for overall and CSS in men by race and then race and region using log-rank tests to detect significant group differences (*p*<0.05). Five-year survival differences for all groups were also calculated using actuarial methods. Multivariable Cox proportional hazards regression models were used to calculate adjusted hazard ratios (aHR) and 95% confidence intervals (CI) for mortality (overall and CSS). Multivariable models included: race (white, black, other), geographic region (West, South, Northeast, Midwest), marital status (single; married or domestic partner; divorced, widowed, or separated), and year of diagnosis (2000–2013) with the largest category in each group as the reference. Age of diagnosis, and census tract level income were continuous variables. Incidence rates and survival analyses were conducted in SEER*Stat and SAS version 9.4 (SAS Institute, Cary, NC).

## Results

### KS incidence

**Fig 1:**
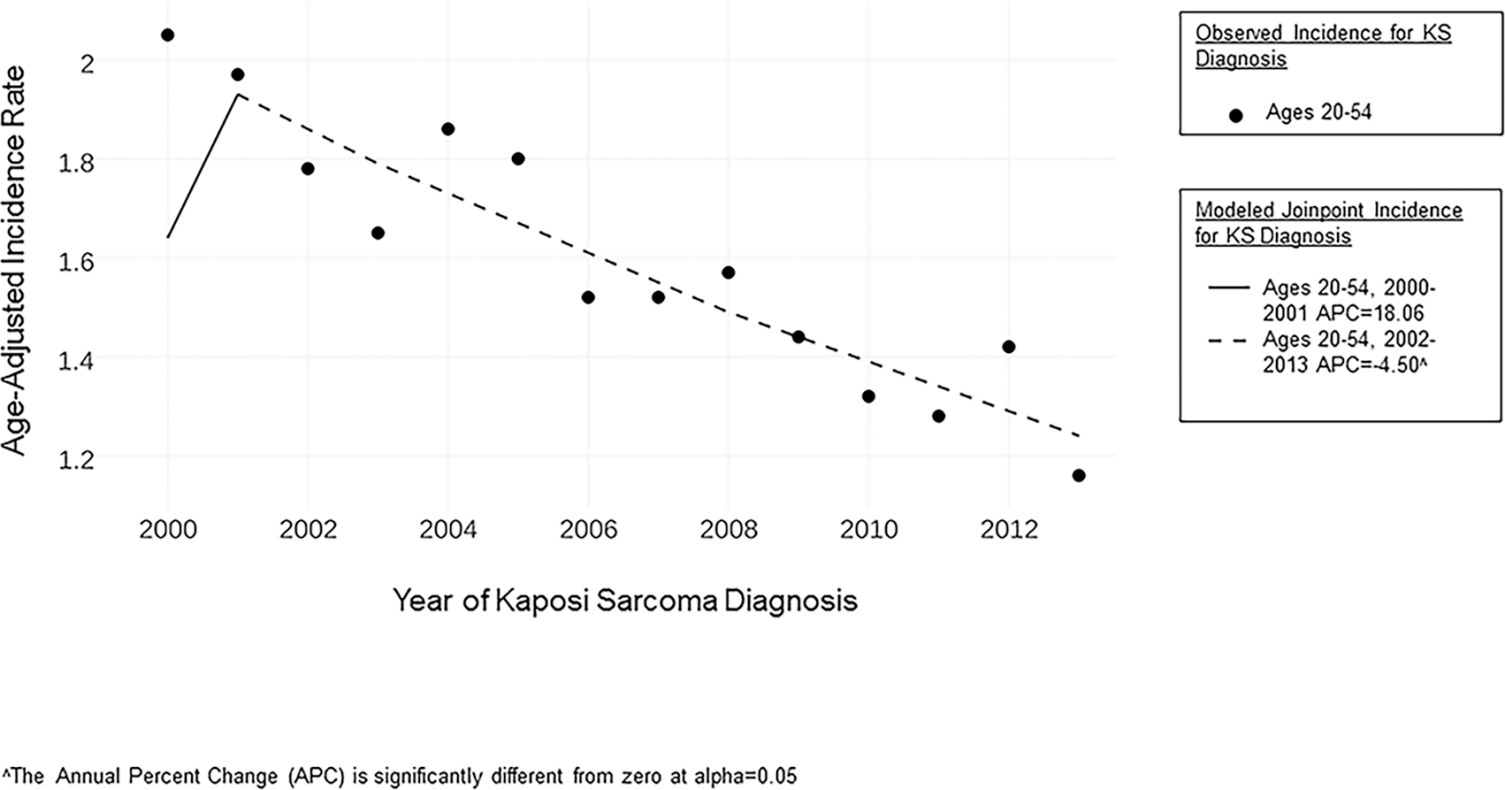
KS incidence in the U.S., 2000–2013, N = 4,455.

From 2000 to 2013, 4455 cases of KS in men younger than 55 years were reported in the SEER database (Table 1). Among cases younger than age 55, 2954 (%) occurred in the West, which includes the largest portion of the general population covered by SEER (Table 1). Fig 1 shows the decline of overall KS incidence from 2001–2013.

**Table 1:** Number of Kaposi sarcoma cases and incidence rates by U.S. region: 2000–2013.

	No. (age-adjusted incidence rate)^a^				
Variable	West	South	Northeast	Midwest	Total
Total	2954 (1.9)	922 (2.0)	402 (1.0)	193 (0.9)	4455
Race					
White	2224 (1.8)	344 (1.0)	215 (0.7)	71 (0.4)	2854
African American	435 (4.3)	563 (5.4)	163 (3.0)	114 (3.5)	1275
Other	157 (0.6)	7 (0.5)	8 (0.2)	2 (0.2)	174
Age					
20–29 years	292 (0.6)	172 (1.3)	48 (0.5)	37 (0.6)	549
30–39 years	1065 (2.3)	387 (2.9)	136 (1.2)	73 (1.2)	1661
40–54 years	1597 (2.4)	363 (1.9)	218 (1.1)	83 (0.8)	2261
Year of diagnosis					
2000	280 (2.5)	65 (2.0)	44 (1.4)	19 (1.0)	408
2001	272 (2.4)	61 (1.9)	39 (1.3)	20 (1.2)	392
2002	235 (2.1)	63 (2.0)	40 (1.3)	15 (0.9)	353
2003	221 (2.0)	60 (1.9)	30 (1.0)	11 (0.7)	322
2004	266 (2.4)	51 (1.6)	37 (1.2)	14 (0.8)	368
2005	231 (2.1)	68 (2.1)	36 (1.2)	14 (0.9)	349
2006	193 (1.7)	56 (1.7)	35 (1.2)	11 (0.7)	295
2007	201 (1.8)	68 (2·1)	25 (0.8)	11 (0.7)	305
2008	199 (1.7)	71 (2.2)	25 (0.8)	16 (1.0)	311
2009	201 (1.7)	62 (1.9)	18 (0.7)	13 (0.9)	294
2010	174 (1.5)	63 (1.9)	20 (0.7)	17 (1.1)	274
2011	156 (1.4)	69 (2.1)	19 (0.6)	10 (0.6)	254
2012	173 (1.5)	99 (3.0)	17 (0.6)	10 (0.7)	299
2013	152 (1.3)	66 (1.9)	18 (0.6)	12 (0.8)	248

^a^Rates are per 100 000 person-years and adjusted to the 2000 US Standard Population.

**Fig 2:**
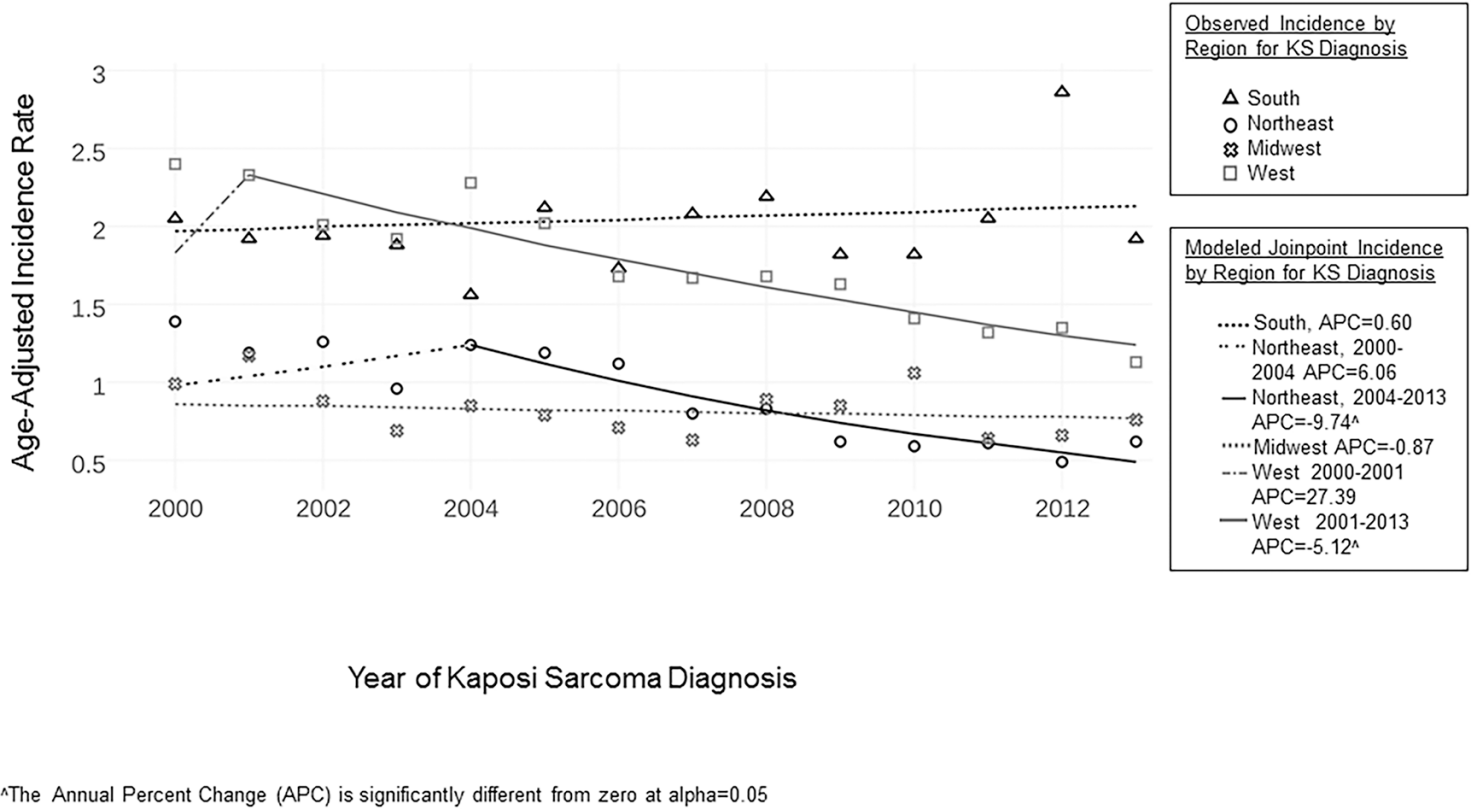
KS incidence in the U.S. by geographic region, 2000–2013.

Fig 2 shows incidence by region from 2000 to 2013. The incidence in the South was 2.0 per 100 000 person-years in 2000 and was generally stable through 2013 with an APC of 0.60, *p* = 0.22. In the West, the incidence decreased from 2.4 per 100,000 person-years in 2000 to 1.1 per 100,000 person-years in 2013, with a joinpoint in 2001 and a significant APC thereafter (2000–2001 APC = 27.39; 2001–2013 APC -5.12, *p* = 0.04). In the Northeast, the incidence was 1.4. per 100,000 person-years in 2000 (APC 6.06) and began a statistically significant decrease in 2004 to 0.6 per 100,000 person-years in 2013 (2000–2004 APC 6.06, *p* = 0.37; 2004–2013 APC = -9.74, *p* = 0.03). Finally, in the Midwest, KS incidence was generally stable with a decreasing overall APC, but the trend did not reach significance (2000–2013 APC -0.87, *p* = 0.56).

**Fig 3:**
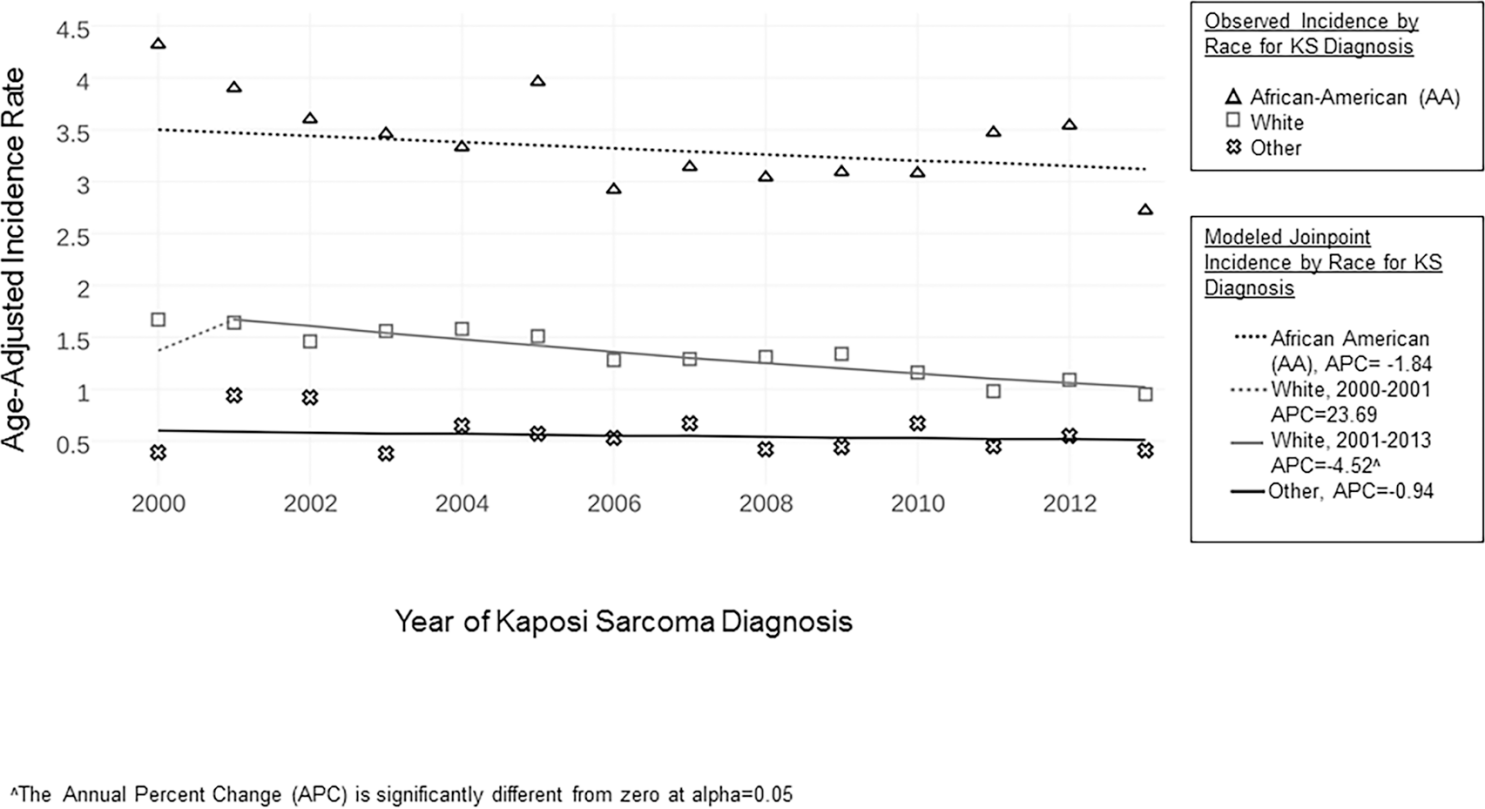
KS incidence in the U.S. by race, 2000–2013.

Fig 3 shows KS incidence by race over time. After 2000, the incidence in white men and other races decreased through 2013. In white men, incidence decreased from 1.7 per 100,000 person-years in 2000 to 0.95 person-years in 2013, with a Joinpoint in 2001 (2001–2013 APC -4.52, *p* = 0.02). In contrast, the incidence for AA men decreased gradually from 4.3 per 100,000 person-years in 2000 to 2.7 per 100,000 person-years in 2013 (2000–2013 APC -1.84, *p* = 0.09). The incidence of KS in other races remained less than half of that of white men at its lowest point in 2013 (2000–2013 APC -0.94, *p* = 0.11). The incidence rate in AA men compared to white men in 2000 was 2.5 fold higher in 2000 and slightly increased to 2.8 fold higher in 2013.

**Fig 4:**
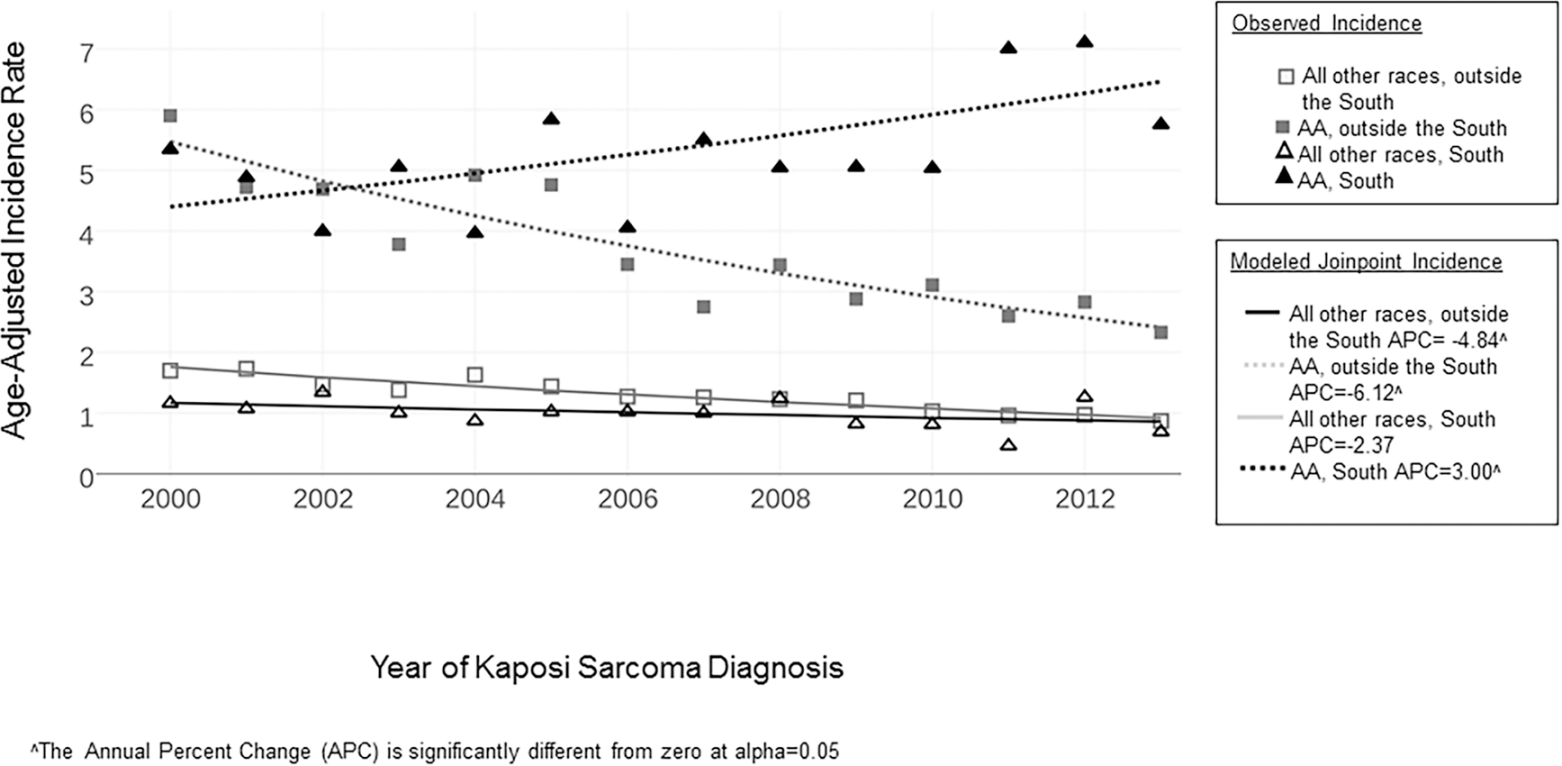
KS incidence in the U.S. by race and geographic, 2000–2013.

To evaluate the potential overlapping effects of race and geographic region, we further evaluated KS incidence in AA men in the South (Fig 4). In this group, KS incidence significantly increased from 5.3 per 100,000 person-years in 2000 to 5.8 per 100,000 person-years in 2013 (2000–2013 APC +3·00, *p* = 0·03). Conversely, the incidence in AA men in regions other than the South (i.e., the West, Northeast, and Midwest combined) significantly decreased from 5.9 per 100,000 person-years in 2000 to 2.3 per 100 000 person-years in 2013 (2000–2013 APC -6.12, *p* = 0·03). In the South, KS incidence in men of races other than AA demonstrated a non-significant decrease over time (2000–2013 APC -2.37, *p* = 0.10). Significant decreases in incidence occurred for non-AA men in regions other than the South (2000–2013 APC -4.8, *p* = 0.05) with an incidence rate in 2013 for this group of 1.0/100,000.

We also conducted a sensitivity analysis evaluating the trends of each of the age groups including those over 55 years, which showed no significant decrease in KS incidence in the over 55 age group by year in contrast to significant changes in all the other age groups (see S1 Fig). With regards to age in the main analysis cohort, KS incidence decreased in the 40–54-year-old age group, KS incidence decreased from 1.1 per 100 000 person-years in 2000 to 0.8 per 100,000 person-years in 2013 (2000–2013 APC -2.47, *p* = 0.03) (S1 Fig). This decrease was even steeper in the 30–39-year-old age group, with a fall in the incidence from 1.6 per 100,000 person-years in 2000 to 0.7 per 100,000 person-years in 2013 (2000–2013 APC -6.10, *p* = 0.02). By contrast, KS incidence in the 20–29-year-old age group demonstrated a non-significant increase from 0.3 per 100,000 person-years in 2000 to 0.5 per 100,000 person-years in 2013 (2000–2013 APC +2·43, *p* = 0·55) (S1 Fig).

### KS survival

Median follow-up time following KS diagnosis was 4.6 years. Table 2 shows multivariable regression models for overall and CSS. AA race compared to White race (aHR 1.49, 95% CI 1.30–1.72), as well as living in the South compared to living in the West (aHR 1.26, 95% CI 1.07–1.48) were associated with higher CSS.

**Table 2:** Crude and adjusted hazard ratios (HR) and 95% confidence intervals (CI) for all-cause and Kaposi sarcoma (KS)-specific mortality among men with KS between 2000 and 2013 (N = 3,749).

	All-cause mortality		KS specific mortality	
	Adjusted HR^a^ (95% CI)	*P*	Adjusted HR^a^ (95% CI)	*P*
Race				
White	1		1	
African American	1.52 (1.34–1.72)	<0·0001	1·49 (1.30–1.72)	<0.0001
Other	0.98 (0.72–1.34)	0·9181	1.05 (0.75–1.46)	0.818
Geographic region				
West	1		1	
South	1.21 (1·04–1·40)	0.0136	1.26 (1.07–1.48)	0.0051
Northeast	1.11 (0.92–1.33)	0.2878	1.10 (0.89–1.35)	0.3662
Midwest	1.20 (0.62–2.24)	0.5689	0.75 (0.31–1.81)	0.5249

^a^All variables adjusted for age of diagnosis (continuous), year of diagnosis (categorical), median family income (continuous), marital status (single; married or domestic partner; divorced, widowed, or separated); only geographic region, race, and are shown.

**Fig 5:**
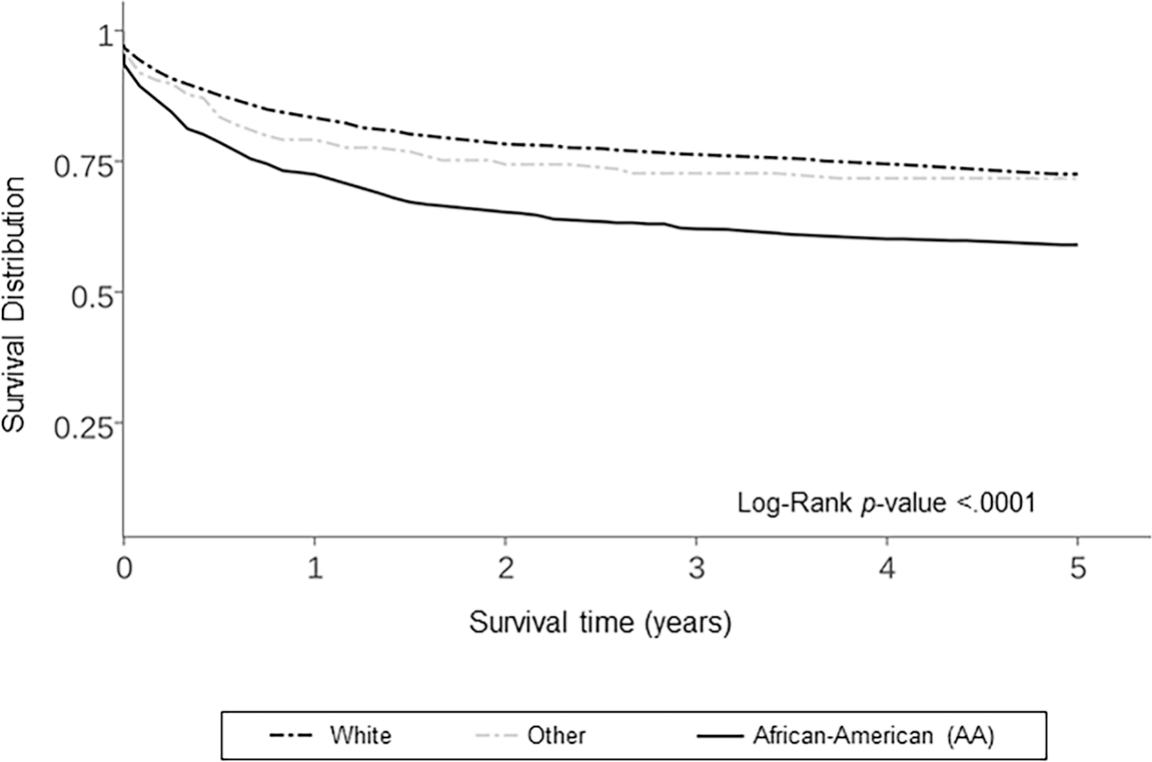
KS survival in the U.S. by Race, 2000–2013, N = 3,793.

**Fig 6:**
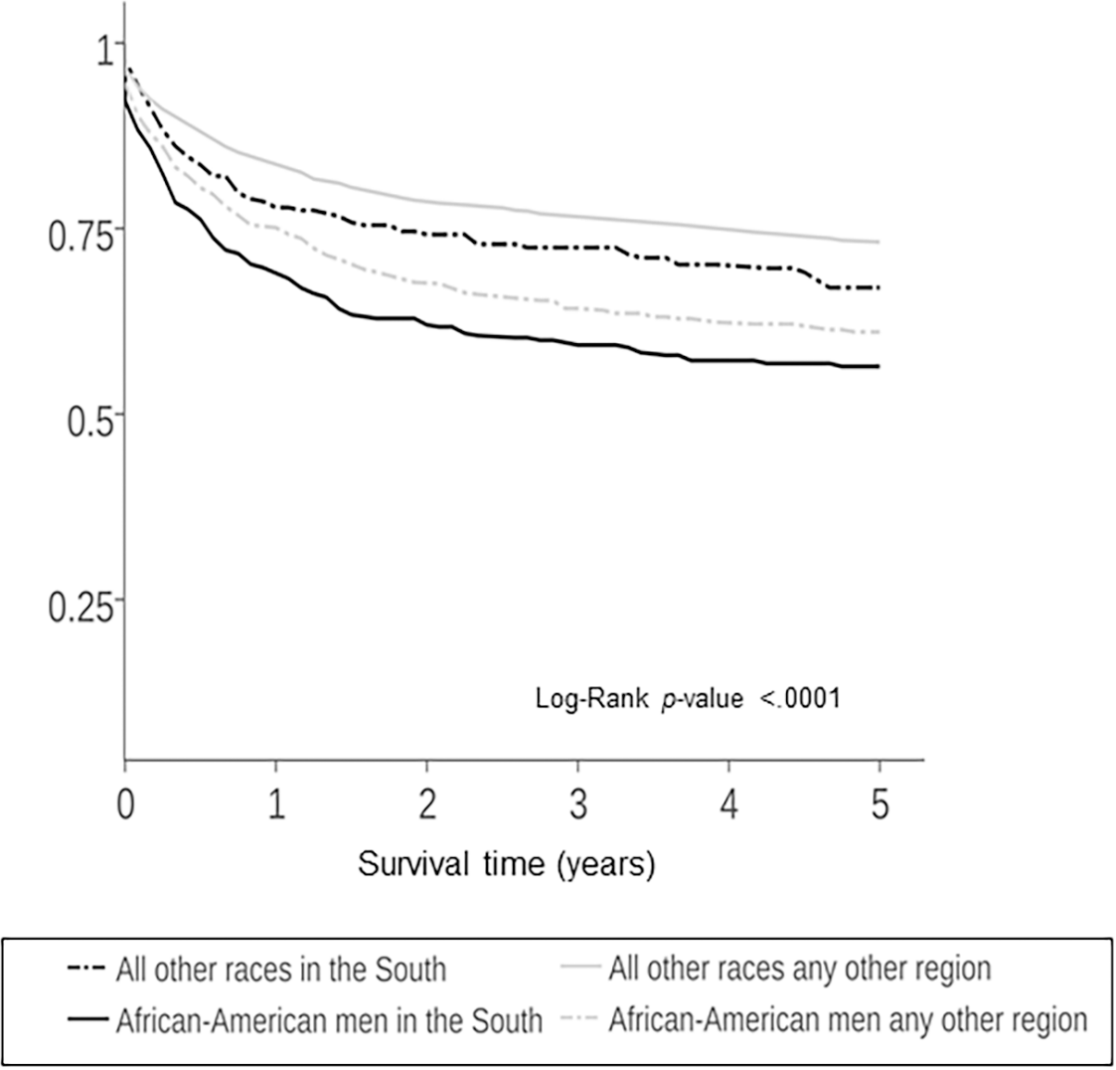
KS survival in the U.S. by Race and Geographic Region, 2000–2013, N = 3,793.

Five year cancer specific survival curves were significantly different (*P*<0.0001) by race (Fig 5). Five year KS cancer-specific survival for AA men was (63.3%), compared with white men (75.5%) and men of other races (74.0%). AA men in the South had lower 5-year overall survival (61.1%) compared to AA men in other regions (64.9%) or men of any other races in any region (Fig 6).

## Discussion

Our findings demonstrate that KS disproportionately affects African Americans, particularly those living in the Southern U.S. This study suggests that the prior reports of the declining KS incidence in the U.S., likely as a result of ART access for HIV-infected individuals, are not evenly distributed across the U.S. population. Populations experiencing the most significant decline in KS incidence are specifically in the Western and Northeastern regions of the U.S. These declines are consistent with declining estimated AIDS incidence in these regions, however, the South continues to have the largest burden of incident AIDS diagnoses, with even a slight increase in incidence in 2013 (S1 Table). In addition, we found that although KS incidence has decreased in non-African Americans groups more broadly across all areas of the U.S., the KS incidence rates for AA nationally has generally been stable between 2000 and 2013, AA in the South experienced a significant increase in KS incidence rates from 2000–2013. In fact, in 2013, the KS incidence rate among AA men in the South was nearly 6 times higher than that of men of all other races in the South (5.8 versus 1.0 per 100,000 person-years, respectively).

Geographic disparities in HIV incidence, prevention and care have been previously documented. In particular, the South has the highest numbers of incident AIDS diagnoses (see S1 Table), highest prevalence of AIDS cases, and most AIDS deaths in the United States[10, 25]. AA in the South also appear to be uniquely affected by KS as evidenced by the significant decline in KS incidence experienced by all other racial groups in the South during that same time period. Thus, it is likely that the differences in KS incidence and mortality may be the result of a confluence of factors related to increasing HIV prevalence, healthcare access, socioeconomic inequality, and possibly HHV-8 epidemiology[26–28].

Furthermore, we found that AA men diagnosed with KS had significantly higher rates of KS cancer specific and all-cause mortality when compared with their white counterparts. Of note, ART has been highly associated with improved survival associated with KS, and thus our findings may be associated with differences in rate ART receipt among AA men in the South [29]. Our findings are consistent with evidence that show AA with HIV have higher all-cause mortality rates compared to other individuals with HIV[30]. In addition, we found that the KS cancer specific 5-year survivals of AA men in the South is lower than all other groups, demonstrating that for AA men with KS and HIV, particularly AA men in the South, KS remains an important cause of death.

In this analysis, we were unable to evaluate the effects of HHV-8 prevalence on differences in KS incidence by geographic region. HHV-8 infection is necessary but insufficient on its own for the development of KS, and HHV-8 prevalence in the United States varies[31]. Studies conducted in the pre-ART/early ART eras found that HHV-8 prevalence is highest among MSM and is positively associated with the number of lifetime sexual partners[32]. HHV-8 prevalence among US MSM remains high in recent estimates, with seroconversion commonly demonstrated in white MSM before the age of 30[33]. Additionally, a meta-analysis of worldwide HIV and HHV-8 coinfection found HIV-positive MSM the most likely to be co-infected of all groups studied (OR 3.95, 95% CI 2.92–5.35)[34]. Of note, there are very few studies of HHV-8 prevalence in AAs, and our findings suggest that an improved understanding of HHV-8 seroprevalence and transmission in young AA men in the South, and among MSM of color in particular is needed.

Our study has some other limitations. Because the SEER registry does not include HIV status, we considered patients with KS who were younger than 55 years at the time of diagnosis to have AIDS-related KS. With this classification, we are not able to specifically evaluate incidence and outcomes for AIDS-related KS in individuals aged 55 years or older by race and region because of limited sample size. However, based on previous validation studies, approximately 94% of cases will be associated with HIV [16]. In addition, individuals with transplant-related KS may also have been included; however, the incidence of transplant related KS remains low[3]. In addition, because of the geographic grouping of SEER sites, the geographic region of the South in our study only consists of two sites (Kentucky, and Georgia), a small percentage of the Southern population. Similarly, the majority of cases were reported from the West, which may have biased the results, however, in spite of the disproportionate number of cases reported in each of the regions, we had enough power to detect trends in the South. Indeed, our findings may be an under-estimate KS incidence rates in the South, given that some of the Southern states with areas of the highest HIV incidence are not included in this particular SEER analysis. Finally, because of limitations in the SEER data we were not able to evaluate the importance of certain KS-associated factors, including HHV-8 seroprevalence, sexual orientation, socioeconomic data, and treatment.

In conclusion, our results indicate widening disparities in KS incidence and survival between AA men and men of other groups in the US, particularly in the Southern U.S. KS morbidity and mortality remain important problems for racial and geographic subsets of the U.S. population. Further research is urgently needed to identify and address the causes of the increasing racial and geographic inequalities in AIDS-related KS disease burden and outcomes.

## Supporting information

S1 Fig KS incidence in the U.S. 2000–2013 by age. (TIF)

S1 Table Estimated numbers of AIDS cases and diagnosis by area of residence-United States, 2002–2013. (DOCX)

## References

[1] Antman K, Chang Y. Kaposi's sarcoma. N Engl J Med. 2000;342(14):1027–38. doi: 10.1056/NEJM200004063421407 10749966 10.1056/NEJM200004063421407

[2] Hiatt KM, Nelson AM, Lichy JH, Fanburg-Smith JC. Classic Kaposi Sarcoma in the United States over the last two decades: a clinicopathologic and molecular study of 438 non-HIV-related Kaposi Sarcoma patients with comparison to HIV-related Kaposi Sarcoma. Modern pathology: an official journal of the United States and Canadian Academy of Pathology, Inc. 2008;21(5):572–82. 10.1038/modpathol.2008.15 18376387

[3] Chaturvedi AK, Engels EA, Pfeiffer RM, Hernandez BY, Xiao W, Kim E, et al Human papillomavirus and rising oropharyngeal cancer incidence in the United States. Journal of clinical oncology: official journal of the American Society of Clinical Oncology. 2011;29(32):4294–301. 21969503 10.1200/JCO.2011.36.4596 PMC3221528

[4] Grulich AE, van Leeuwen MT, Falster MO, Vajdic CM. Incidence of cancers in people with HIV/AIDS compared with immunosuppressed transplant recipients: a meta-analysis. Lancet (London, England). 2007;370(9581):59–67. 10.1016/S0140-6736(07)61050-2 17617273

[5] Armstrong AW, Lam KH, Chase EP. Epidemiology of classic and AIDS-related Kaposi's sarcoma in the USA: incidence, survival, and geographical distribution from 1975 to 2005. Epidemiol Infect. 2013;141(1):200–6. doi: 10.1017/S0950268812000325 22404880 10.1017/S0950268812000325 PMC9152052

[6] Eltom MA, Jemal A, Mbulaiteye SM, Devesa SS, Biggar RJ. Trends in Kaposi's sarcoma and non-Hodgkin's lymphoma incidence in the United States from 1973 through 1998. J Natl Cancer Inst. 2002;94(16):1204–10. 12189223 10.1093/jnci/94.16.1204

[7] Beral V, Peterman TA, Berkelman RL, Jaffe HW. Kaposi's sarcoma among persons with AIDS: a sexually transmitted infection? Lancet. 1990;335(8682):123–8. 1967430 10.1016/0140-6736(90)90001-l

[8] Boscoe FP, Johnson CJ, Sherman RL, Stinchcomb DG, Lin G, Henry KA. The relationship between area poverty rate and site-specific cancer incidence in the United States. Cancer. 2014;120(14):2191–8. doi: 10.1002/cncr.28632 24866103 10.1002/cncr.28632 PMC4232004

[9] NCHHSTP. Persistent Disparities Prolong HIV Epidemic. Despite progress, persistent disparities prolong HIV epidemic among African Americans: CDC; 2014 [cited 2016 February 13]. Available from: http://www.cdc.gov/nchhstp/newsroom/2016/nbhaad-press-release.html.

[10] NHPC Press Release: Southern States. Southern States Lag Behind Rest of Nation in HIV Treatment, Testing 2015. Available from: http://www.cdc.gov/nchhstp/newsroom/2015/nhpc-press-release-southern-states.html.

[11] SEER*Stat Database: Incidence—SEER 17 Regs Research Data [Internet]. 2014. Available from: www.seer.cancer.gov.

[12] Surveillance, Epidemiology, and End Results (SEER) Program (www.seer.cancer.gov) SEER*Stat Database: Incidence—SEER 17 Regs Research Data, Nov 2014 Sub (1973–2012) <Katrina/Rita Population Adjustment>—Linked To County Attributes—Total U.S., 1969–2013 Counties, National Cancer Institute, DCCPS, Surveillance Research Program, Surveillance Systems Branch, released April 2015, based on the November 2014 submission.

[13] Zippin C, Lum D, Hankey BF. Completeness of hospital cancer case reporting from the SEER Program of the National Cancer Institute. Cancer. 1995;76(11):2343–50. 8635041 10.1002/1097-0142(19951201)76:11<2343::aid-cncr2820761124>3.0.co;2-#

[14] Di Lorenzo G. Update on classic Kaposi sarcoma therapy: new look at an old disease. Crit Rev Oncol Hematol. 2008;68(3):242–9. doi: 10.1016/j.critrevonc.2008.06.007 18657433 10.1016/j.critrevonc.2008.06.007

[15] Dal Maso L, Polesel J, Ascoli V, Zambon P, Budroni M, Ferretti S, et al Classic Kaposi's sarcoma in Italy, 1985–1998. Br J Cancer. 2005;92(1):188–93. doi: 10.1038/sj.bjc.6602265 15570306 10.1038/sj.bjc.6602265 PMC2361748

[16] Shiels MS, Pfeiffer RM, Hall HI, Li J, Goedert JJ, Morton LM, et al Proportions of Kaposi sarcoma, selected non-Hodgkin lymphomas, and cervical cancer in the United States occurring in persons with AIDS, 1980–2007. JAMA. 2011;305(14):1450–9. doi: 10.1001/jama.2011.396 21486978 10.1001/jama.2011.396 PMC3909038

[17] Statistical Methodology and Applications Branch, Surveillance Research Program. Joinpoint Regression Program: National Cancer Institute; 2016.

[18] Kim HJ, Fay MP, Feuer EJ, Midthune DN. Permutation tests for joinpoint regression with applications to cancer rates. Stat Med. 2000;19(3):335–51. 10649300 10.1002/(sici)1097-0258(20000215)19:3<335::aid-sim336>3.0.co;2-z

[19] Howlader N, Ries LA, Mariotto AB, Reichman ME, Ruhl J, Cronin KA. Improved estimates of cancer-specific survival rates from population-based data. Journal of the National Cancer Institute. 2010;102(20):1584–98. doi: 10.1093/jnci/djq366 20937991 10.1093/jnci/djq366 PMC2957430

[20] Marcus JL, Chao C, Leyden WA, Xu L, Yu J, Horberg MA, et al Survival among HIV-infected and HIV-uninfected individuals with common non-AIDS-defining cancers. Cancer epidemiology, biomarkers & prevention: a publication of the American Association for Cancer Research, cosponsored by the American Society of Preventive Oncology. 2015;24(8):1167–73. 10.1158/1055-9965.EPI-14-1079 PMC4526437 25713023

[21] Simard EP, Pfeiffer RM, Engels EA. Mortality due to cancer among people with AIDS: a novel approach using registry-linkage data and population attributable risk methods. AIDS (London, England). 2012;26(10):1311–8. 10.1097/QAD.0b013e328353f38e PMC3377813 22472857

[22] Centers for Disease Control and Prevention. Cases of HIV infection and AIDS in the United States and dependent areas, by race/ethnicity, 2002–2006, vol 13 [Internet]. Atlanta; 2006. [cited 2017 Jun 27]. Available from: http://www.cdc.gov/hiv/topics/surveillance/resources/reports/2008supp_vol13no1/.

[23] Centers for Disease Control and Prevention. HIV Surveillance Report, 2009; vol. 21[Internet]. Atlanta; 2011 [cited 2017 Jun 27]. Available from: http://www.cdc.gov/hiv/topics/surveillance/resources/reports/.

[24] Centers for Disease Control and Prevention. HIV Surveillance Report, 2013; vol. 25 [Internet]. Atlanta; 2015 [cited 2017 June 27]. Available from: http://www.cdc.gov/hiv/library/reports/surveillance/.

[25] CDC. HIV and AIDS in the United States by Geographic Distribution: CDC; 2015 [cited 2016 February 14]. Available from: http://www.cdc.gov/hiv/statistics/overview/geographicdistribution.html.

[26] Siegel RL, Miller KD, Jemal A. Cancer statistics, 2016. CA Cancer J Clin. 2016;66(1):7–30. doi: 10.3322/caac.21332 26742998 10.3322/caac.21332

[27] Ward E, Jemal A, Cokkinides V, Singh GK, Cardinez C, Ghafoor A, et al Cancer disparities by race/ethnicity and socioeconomic status. CA Cancer J Clin. 2004;54(2):78–93. 15061598 10.3322/canjclin.54.2.78

[28] Dasgupta S, Oster AM, Li J, Hall HI, DPE. Disparities in Consistent Retention in HIV Care—11 States and the District of Columbia, 2011–2013. MMWR Morb Mortal Wkly Rep. 2016;65(4):77–82. doi: 10.15585/mmwr.mm6504a2 26844978 10.15585/mmwr.mm6504a2

[29] Gbabe OF, Okwundu CI, Dedicoat D, Freeman EE. Treatment of severe or progressive Kaposi's sarcoma in HIV-infected adults. Cochrane Database Syst Rev. 2014;(9):CD003256 doi: 10.1002/14651858.CD003256.pub2 25313415 10.1002/14651858.CD003256.pub2

[30] Siddiqi AA HX, Hall HI. Mortality Among Blacks or African Americans with HIV Infection—United States, 2008–2012: CDC; 2015 [cited 2016 March 3]. Available from: http://www.cdc.gov/mmwr/preview/mmwrhtml/mm6404a2.htm. PMC4584853 25654607

[31] Uldrick TS, Whitby D. Update on KSHV epidemiology, Kaposi Sarcoma pathogenesis, and treatment of Kaposi Sarcoma. Cancer Lett. 2011;305(2):150–62. doi: 10.1016/j.canlet.2011.02.006 21377267 10.1016/j.canlet.2011.02.006 PMC3085592

[32] Smith NA, Sabin CA, Gopal R, Bourboulia D, Labbet W, Boshoff C, et al Serologic evidence of human herpesvirus 8 transmission by homosexual but not heterosexual sex. J Infect Dis. 1999;180(3):600–6. doi: 10.1086/314926 10438345 10.1086/314926

[33] Labo N, Miley W, Benson CA, Campbell TB, Whitby D. Epidemiology of Kaposi's sarcoma-associated herpesvirus in HIV-1-infected US persons in the era of combination antiretroviral therapy. AIDS. 2015;29(10):1217–25. doi: 10.1097/QAD.0000000000000682 26035321 10.1097/QAD.0000000000000682 PMC6680245

[34] Rohner E, Wyss N, Heg Z, Faralli Z, Mbulaiteye SM, Novak U, et al HIV and human herpesvirus 8 co-infection across the globe: Systematic review and meta-analysis. International journal of cancer. 2016;138(1):45–54. doi: 10.1002/ijc.29687 26175054 10.1002/ijc.29687 PMC4607648

