# Supplementary figures and images for: Disparities in Kaposi sarcoma incidence and survival in the United States: 2000-2013
Source: PLoS One. 2017 Aug 22;12(8):e0182750. doi: 10.1371/journal.pone.0182750 (PMC5567503; doi:10.1371/journal.pone.0182750)

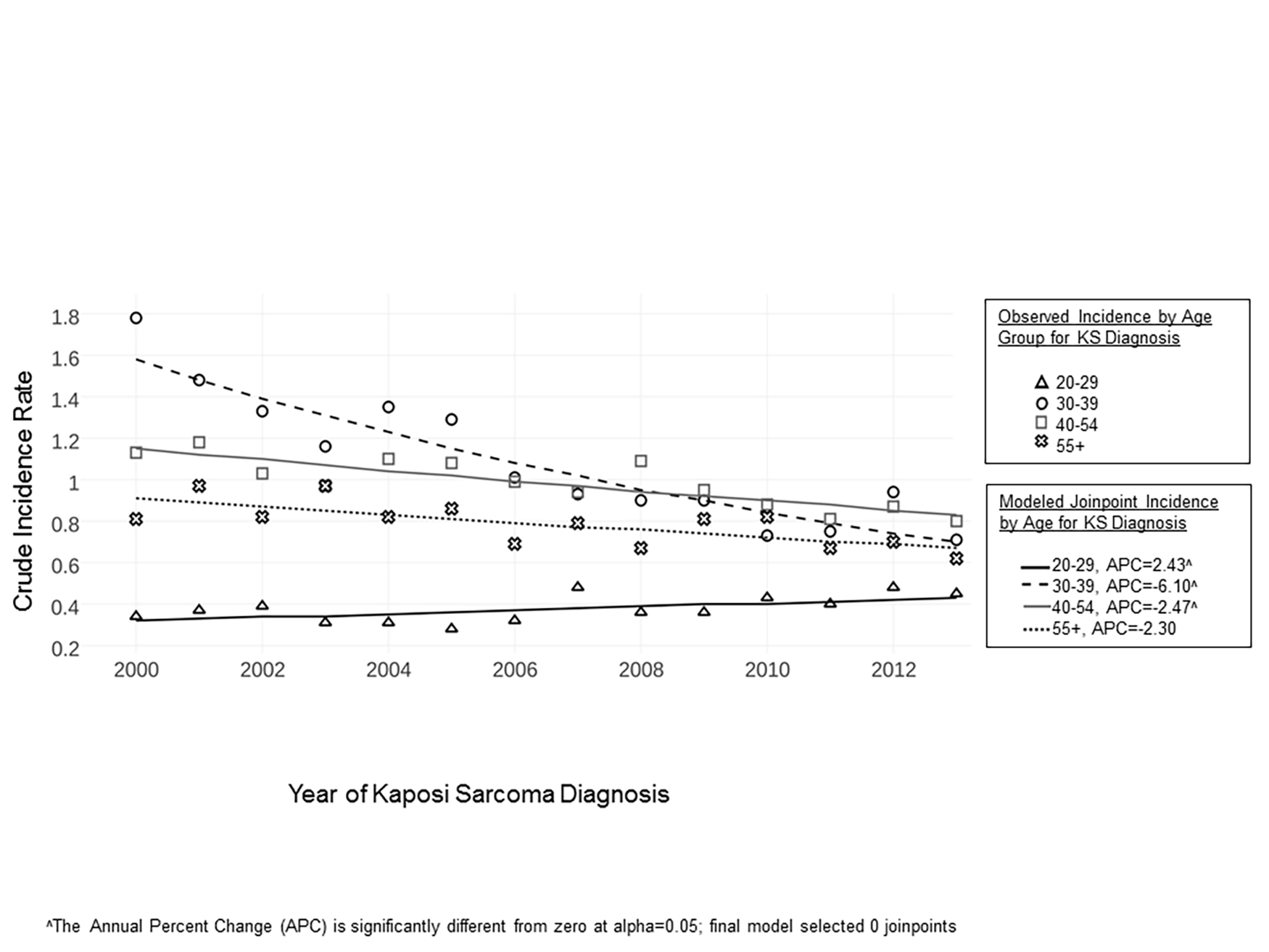

Supplement: S1 Fig — (TIF) [file pone.0182750.s001.tif]
