# Supplementary material for: Disparities in Kaposi sarcoma incidence and survival in the United States: 2000-2013
Source: PLoS One. 2017 Aug 22;12(8):e0182750. doi: 10.1371/journal.pone.0182750 (PMC5567503; doi:10.1371/journal.pone.0182750)
Supplement: S1 Table — (DOCX) [file pone.0182750.s002.docx]

| S1 Table. Estimated numbers of AIDS cases and diagnosis by area of residence-United States, 2002-2013 | | | | | | | | |
| --- | --- | --- | --- | --- | --- | --- | --- | --- |
|  | Region | | | | | | | |
|  | Northeast | | Midwest | | South | | West | |
| Year | No.* | Rate* | No.* | Rate* | No.* | Rate* | No.* | Rate* |
| 2002^a^ | 10,095 | N/A | 4,133 | N/A | 17,243 | N/A | 6,721 | N/A |
| 2003^a^ | 10,346 | N/A | 4,239 | N/A | 17,701 | N/A | 6,343 | N/A |
| 2004^a^ | 9,419 | N/A | 4,089 | N/A | 18,379 | N/A | 6,000 | N/A |
| 2005^a^ | 9,414 | N/A | 4,373 | N/A | 17,025 | N/A | 5,894 | N/A |
| 2006^b^ | 9,369 | 17.1 | 4,154 | 6.3 | 16,453 | 15.1 | 6,174 | 9.0 |
| 2007^b^ | 9,082 | 16.5 | 4,006 | 6.0 | 16,383 | 14.8 | 5,964 | 8.5 |
| 2008^b^ | 8,064 | 14.6 | 4,218 | 6.3 | 16,506 | 14.7 | 5,967 | 8.4 |
| 2009^c^ | 6,750  8,171 | 12.2  14.8 | 3,714  4,394 | 5.6  6.6 | 15,036  15,806 | 13.3  13.9 | 5,761  5,875 | 8.0  8.2 |
| 2010^c^ | 5,923 | 10.7 | 3,456 | 5.2 | 14,183 | 12.3 | 5,054 | 7.0 |
| 2011^c^ | 5,449 | 9.8 | 3,356 | 5.0 | 13,466 | 11.6 | 4,484 | 6.2 |
| 2012^c^ | 5,173 | 9.3 | 3,283 | 4.9 | 13,109 | 11.2 | 4,408 | 6.0 |
| 2013^c^ | 4,872 | 8.7 | 3,221 | 4.8 | 14,345 | 12.1 | 4,251 | 5.7 |
| Cumulative^c,d^ | 352,167 | N/A | 126,372 | N/A | 477,964 | N/A | 237,536 | N/A |
| a, b, c are refs [22 – 24] *Estimated | | | | | | | | |
